# Supplementary material for: Pulmonary pericytes regulate lung morphogenesis
Source: Nat Commun. 2018 Jun 22;9:2448. doi: 10.1038/s41467-018-04913-2 (PMC6015030; doi:10.1038/s41467-018-04913-2)
Supplement: Supplementary file 1 — Supplementary information [file 41467_2018_4913_MOESM1_ESM.pdf]

Supplementary Information

Pulmonary pericytes regulate lung morphogenesis

Kato et al

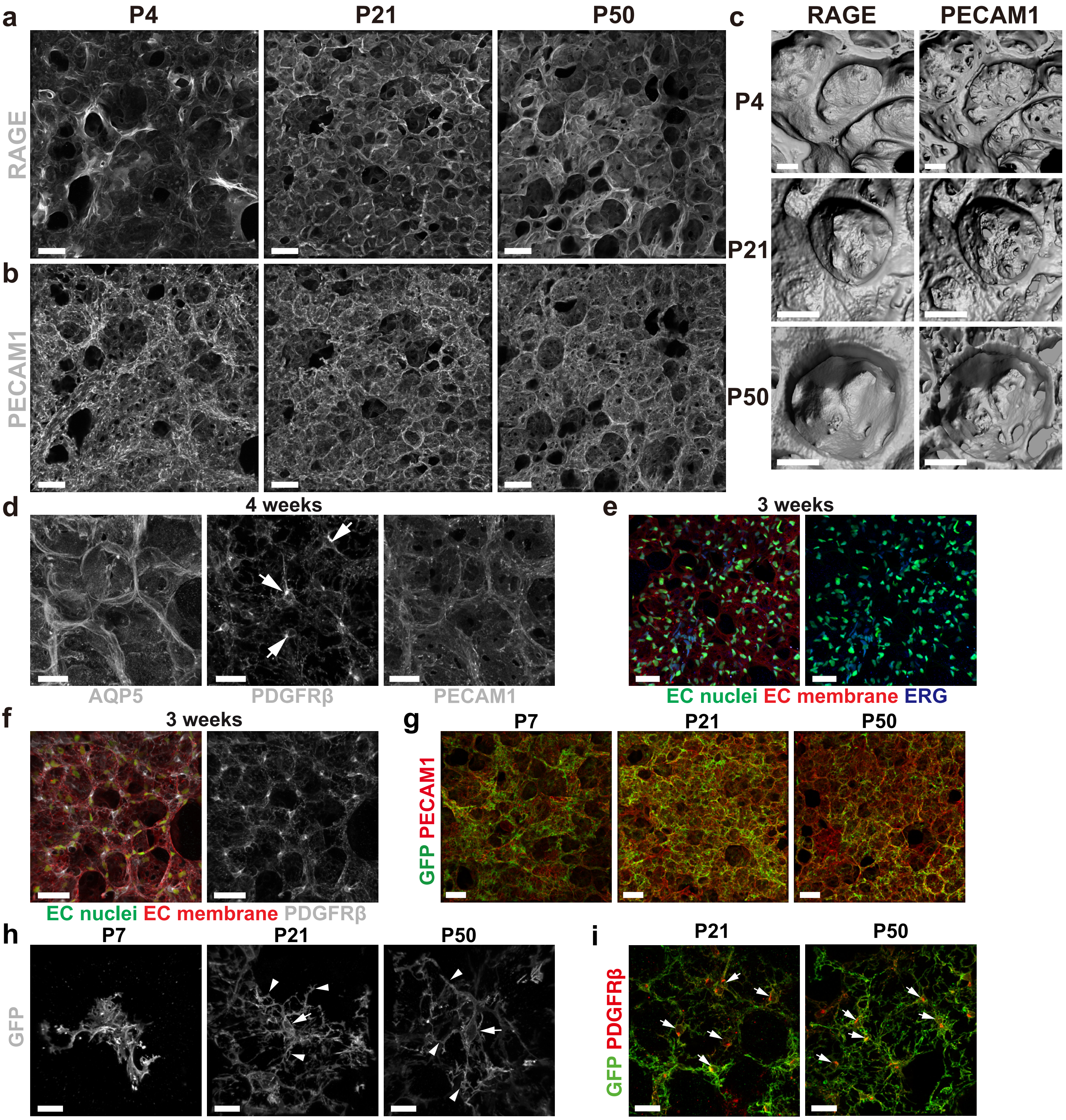

Supplementary Figure 1 Characterisation of *Pdgfrb(BAC)-CreERT2* lungs

(**a**, **b**) Three-dimensional (3D) reconstruction of confocal images showing RAGE-stained type 1 alveolar epithelial cells (**a**) and PECAM1-stained pulmonary ECs (**b**) at the indicated stages. Scale bar, 50μm.

(**c**) 3D reconstruction surface images showing alveolar and capillary organisation at the indicated stages. Scale bar, 20μm.

(**d**) Separated channels of images shown in Fig. 1a. Arrows indicate pericytes. Scale bar, 15μm.

(**e**, **f**) 3D reconstruction of confocal images from 3-week-old *Cdh5-mT/nG* lungs (EC plasma membrane in red and nuclei in green) immunostained for ERG (blue, **e**) or PDGFRβ (white, **f**). Note that all GFP+ EC nuclei are also positive for ERG. Scale bar, 30μm.

(**g**) 3D reconstruction confocal images of lungs from *Pdgfrb(BAC)-CreERT2 R26-mT/mG* mice at the indicated stages after treatment with tamoxifen from P1 to P3. Pulmonary PCs are visualized by GFP (green) and ECs by PECAM1 immunostaining (red). Scale bar, 50μm.

(**h**) High magnification images of *Pdgfrb(BAC)-CreERT2 R26-mT/mG* lung sections from mice at the indicated stages. A low dose of 4-hydroxytamoxifen was used at P7 to visualize single pericytes. Scale bar, 10μm.

(**i**) High magnification images of *Pdgfrb(BAC)-CreERT2 R26-mT/mG* lung sections at P21 and P50. GFP signal is confined to PDGFRβ+ pericytes (arrows). Scale bar, 20μm.

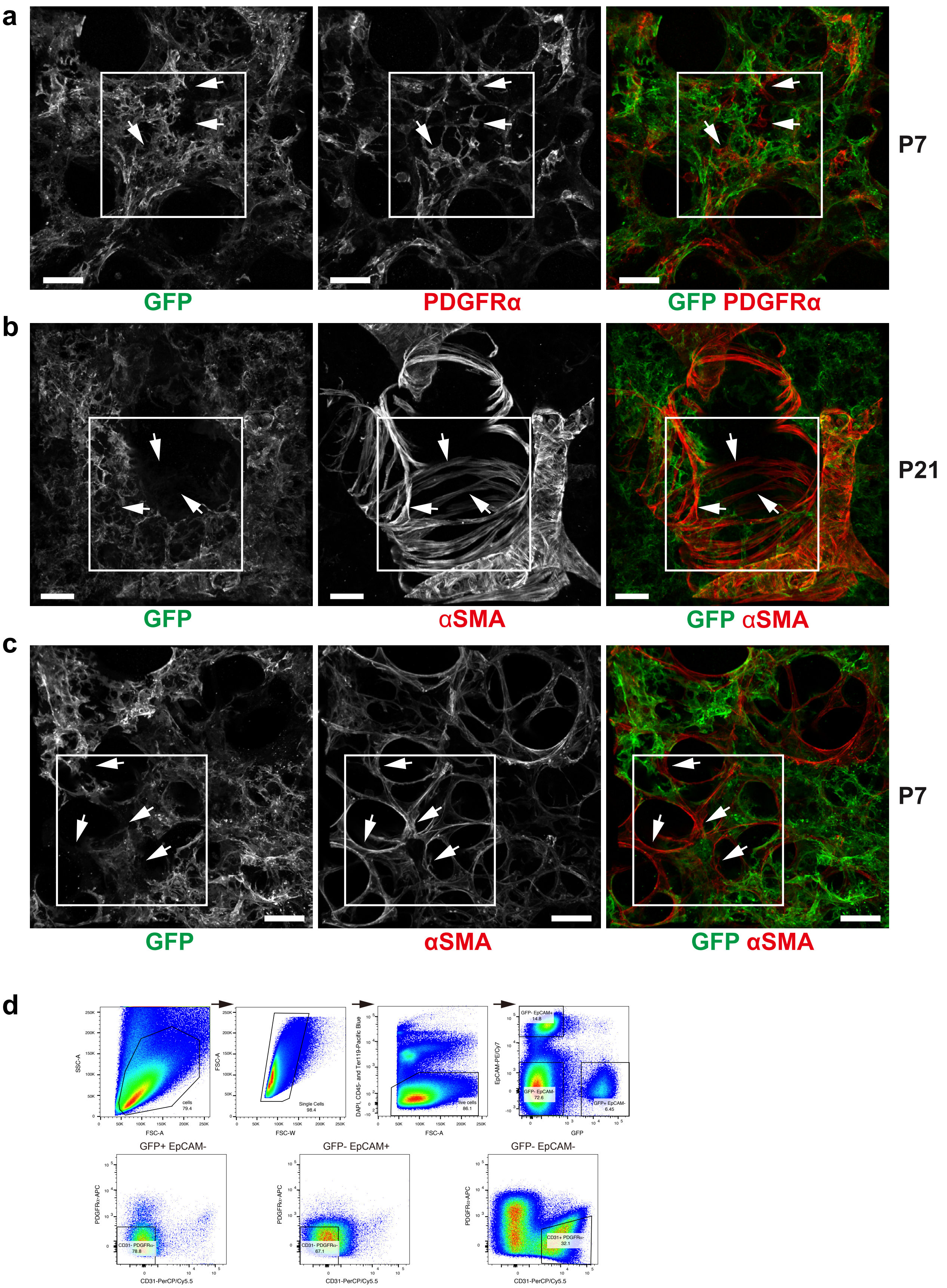

Supplementary Figure 2 Characterization of *Pdgfrb(BAC)-CreERT2* transgenic mice

(**a**-**c**) Wide field view images of panels shown in Fig. 1i-k. Scale bar, 30μm (**a**, **c**) and 50μm (**b**).

(**d**) Gating strategy for the purification of three subpopulations from *Pdgfrb(BAC)-CreERT2 R26-mTmG* lung tissue by flow cytometry. After initial FSC/SSC gating and doublet exclusion, live cells were gated as DAPI- CD45- Ter119-, and then subdivided in two steps into (i) GFP+ EpCAM- CD31- CD140a-, (ii) GFP- EpCAM+ CD31- CD140a- and (iii) GFP- EpCAM- CD31+ CD140a- fractions.

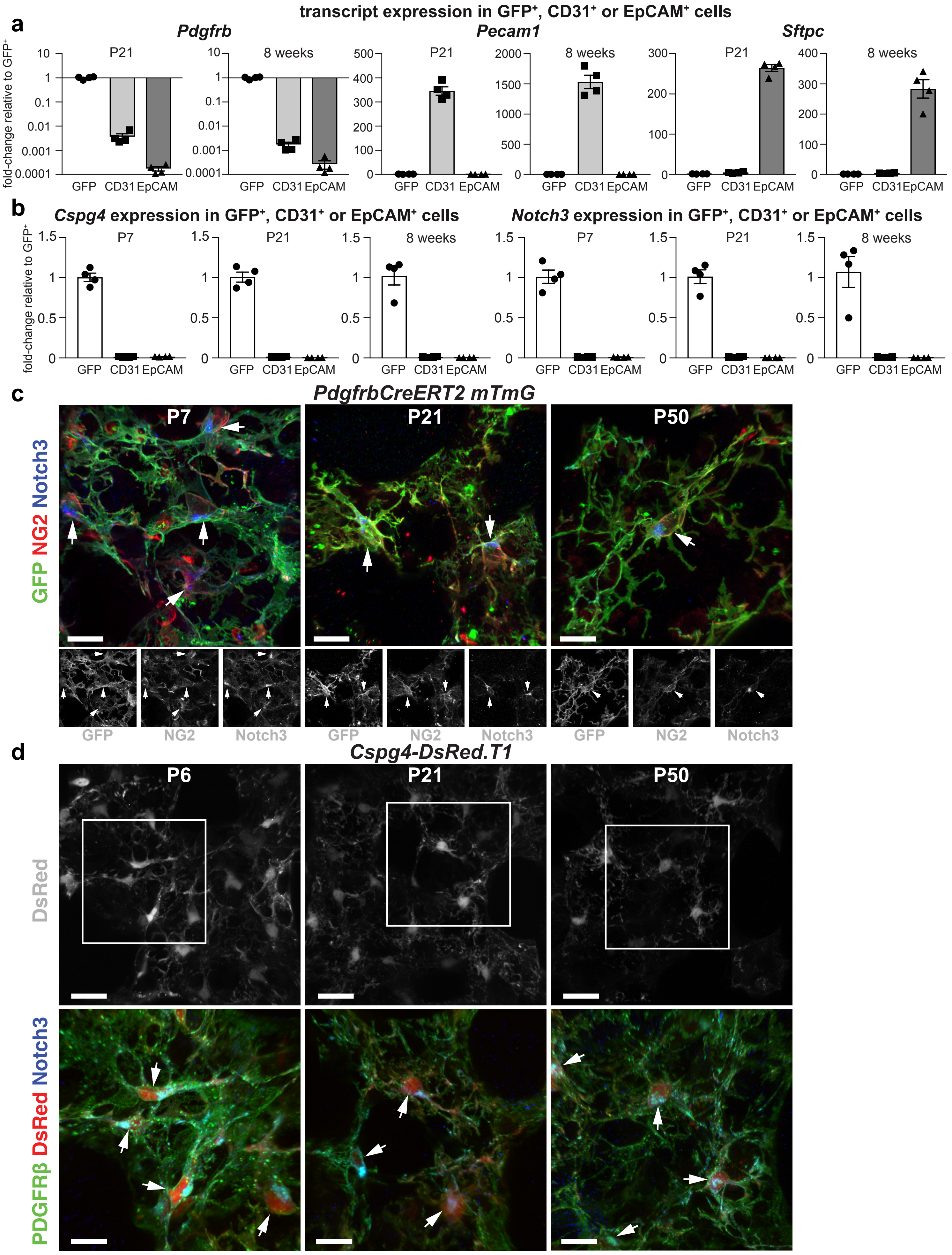
a
transcript expression in GFP⁺, CD31⁺ or EpCAM⁺ cells
Pdgfrb
Pecam1
Sftpc
fold-change relative to GFP⁺
P21
8 weeks
GFP CD31 EpCAM
b
Cspg4 expression in GFP⁺, CD31⁺ or EpCAM⁺ cells
Notch3 expression in GFP⁺, CD31⁺ or EpCAM⁺ cells
P7
P21
8 weeks
c
PdgfrbCreERT2 mTmG
P7
P21
P50
GFP NG2 Notch3
d
Cspg4-DsRed.T1
P6
P21
P50
DsRed
PDGFRβ DsRed Notch3

Supplementary Figure 3 Analysis of pericyte markers in *Pdgfrb(BAC)-CreERT2* lungs

(a) RT-qPCR analysis of the markers *Pdgfrb* (pericytes), *Pecam1* (endothelium) and *Sftpc* (epithelium) expression in freshly sorted lung GFP+, CD31+ or EpCAM+ cells from P21 and 8-week-old *Pdgfrb(BAC)-CreERT2 R26-mT/mG* mice. Data represents mean± s.e.m. (n=4 mice).

(b) RT-qPCR analysis of *Cspg4* and *Notch3* expression in freshly sorted GFP+, CD31+ or EpCAM+ cells from P7, P21 and 8-week-old *Pdgfrb(BAC)-CreERT2 R26-mT/mG* lungs. Data represents mean± s.e.m. (n=4 mice).

(c) High magnification images of GFP (green), NG2 (red) and Notch3 (blue) staining in lung sections from *Pdgfrb(BAC)-CreERT2 R26-mT/mG* mice at the indicated stages. Bottom panels show separated channels of the image above. Scale bar, 10μm.

(d) High magnification images of PDGFRβ (green), DsRed (white/red) and Notch3 (blue) staining in sections from *Cspg4-DsRed.T1* lungs at the indicated stages. Bottom panels show higher magnification of corresponding insets above. Scale bar, 20μm (top panels) and 10μm (bottom).

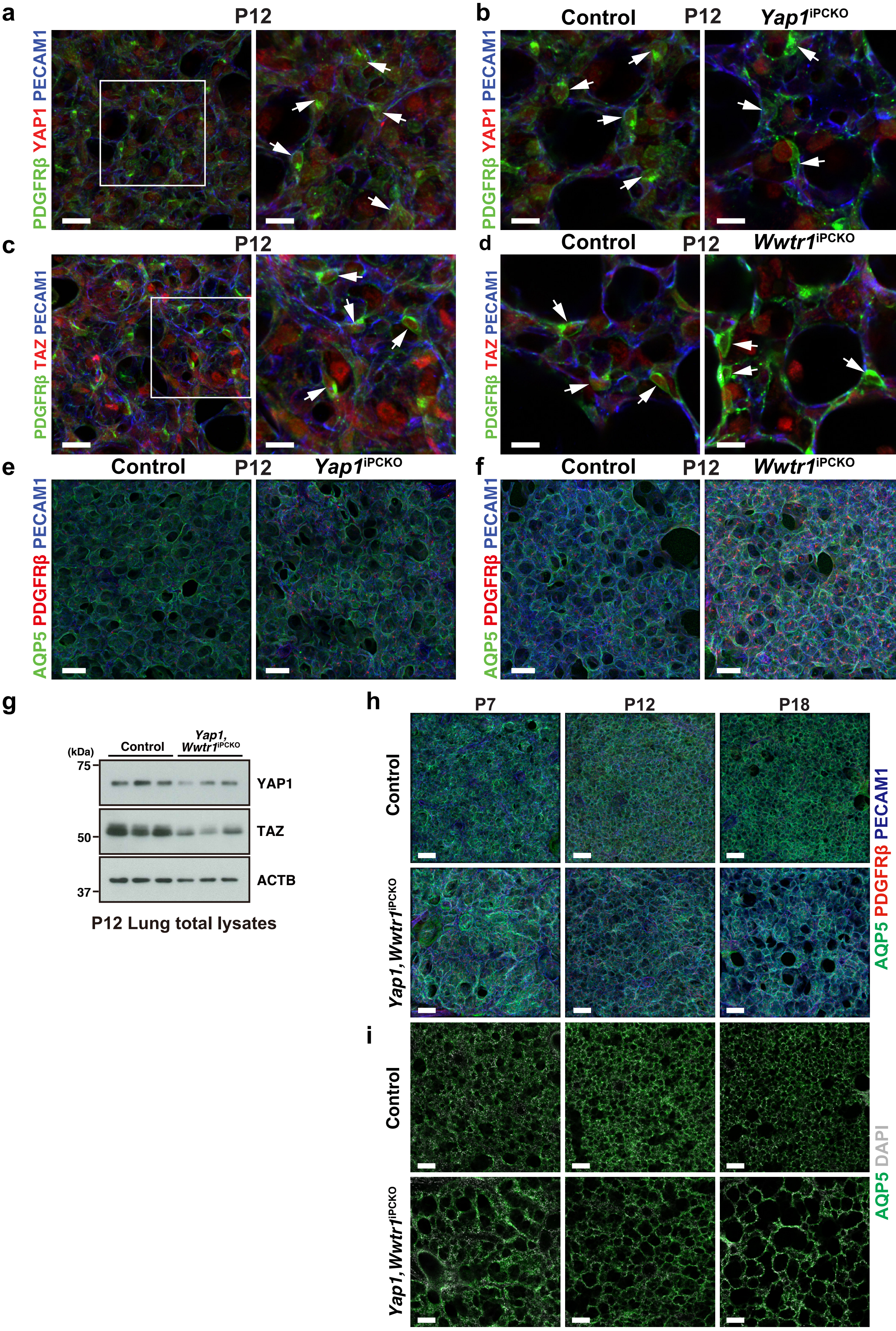
a
P12
PDGFRβ YAP1 PECAM1
b
Control
P12
Yap1iPCKO
PDGFRβ YAP1 PECAM1
c
P12
PDGFRβ TAZ PECAM1
d
Control
P12
Wwtr1iPCKO
PDGFRβ TAZ PECAM1
e
Control
P12
Yap1iPCKO
AQP5 PDGFRβ PECAM1
f
Control
P12
Wwtr1iPCKO
AQP5 PDGFRβ PECAM1
g
(kDa)
Control
Yap1, Wwtr1iPCKO
75
50
37
YAP1
TAZ
ACTB
P12 Lung total lysates
h
P7
P12
P18
Control
Yap1, Wwtr1iPCKO
AQP5 PDGFRβ PECAM1
i
Control
Yap1, Wwtr1iPCKO
AQP5 DAPI

Supplementary Figure 4 Expression of YAP1 and TAZ in lung

(a) Three dimensional reconstruction confocal images of P12 lungs stained for PDGFRβ (green), YAP1 (red) and PECAM1 (blue). Panels on the right show higher magnification of corresponding insets. Arrows indicate YAP1 expression in pericytes. Scale bar, 20μm (left panel) and 10μm (right).

(b) High magnification thin optical section view of confocal images showing the depletion of YAP1 protein in P12 *Yap1*^{iPCKO} lung. Arrows indicate pericytes. Scale bar, 10μm.

(c) 3D reconstruction confocal images of P12 lungs stained for PDGFRβ (green), TAZ (red) and PECAM1 (blue). Panels on the right shows higher magnification of corresponding insets. Arrows indicate TAZ+ pericytes. Scale bar, 20μm (left panel) and 10μm (right).

cells. Arrows indicate pericytes. Scale bar, 10μm.

(e, f) 3D reconstruction of confocal images from P12 *Yap1*^{iPCKO} (**e**) or *Wwtr1*^{iPCKO} (**f**) and (red) and PECAM1 (blue). Scale bar, 50μm.

(g) Western blot analysis of YAP1 and TAZ in P12 *Yap1*,*Wwtr1*^{iPCKO} and littermate control total lung lysates (n=3 mice). Molecular weight marker (kDa) is indicated.

(h, i) Wide field view of maximum intensity projections (**h**) or single optical section views (**i**) (red) and PECAM1 (blue) (**h**) or AQP5 (green) and DAPI (white) (**i**) at the indicated stages. Scale bar, 100μm.

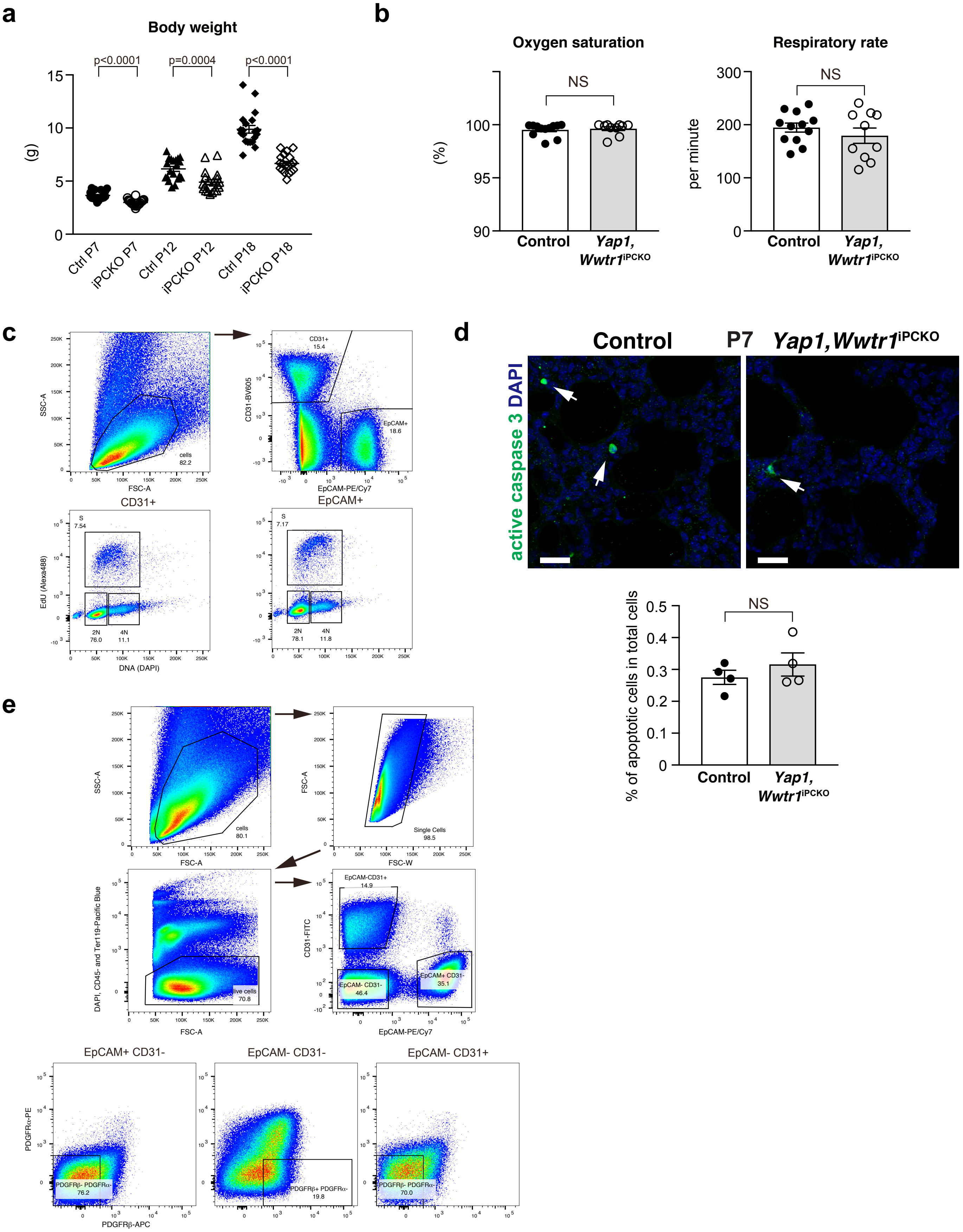
a
Body weight
p<0.0001
p=0.0004
p<0.0001
(g)
15
10
5
0
Ctrl P7
iPCKO P7
Ctrl P12
iPCKO P12
Ctrl P18
iPCKO P18
b
Oxygen saturation
NS
(%)
100
95
90
Control
Yap1, Wwtr1iPCKO
Respiratory rate
NS
per minute
300
200
100
0
Control
Yap1, Wwtr1iPCKO
c
SSC-A
FSC-A
cells 82.2
CD31-BV605
EpCAM-PE/Cy7
CD31+ 15.4
EpCAM+ 18.6
CD31+
EpCAM+
EdU (Alexa488)
DNA (DAPI)
S 7.54
2N 76.0
4N 11.1
S 7.17
2N 78.1
4N 11.8
d
Control
P7
Yap1,Wwtr1iPCKO
active caspase 3 DAPI
% of apoptotic cells in total cells
NS
0.5
0.4
0.3
0.2
0.1
0
Control
Yap1, Wwtr1iPCKO
e
SSC-A
FSC-A
cells 80.1
FSC-A
FSC-W
Single Cells 98.5
DAPI, CD45- and Ter119-Pacific Blue
FSC-A
live cells 70.8
CD31-FITC
EpCAM-PE/Cy7
EpCAM-CD31+ 14.9
EpCAM- CD31- 46.4
EpCAM+ CD31- 35.1
EpCAM+ CD31-
EpCAM- CD31-
EpCAM- CD31+
PDGFRα-PE
PDGFRβ-APC
PDGFRβ- PDGFRα- 76.2
PDGFRβ+ PDGFRα- 19.8
PDGFRβ- PDGFRα- 70.0

Supplementary Figure 5 Physiological analysis of PC-specific *Yap1,Wwtr1* mutants

(**a**) Quantitation of body weight in *Yap1*,*Wwtr1*^{iPCKO} and littermate control mice at the indicated stages. Data represents mean± s.e.m. (n=20 mice, two-tailed unpaired t-test).

(**b**) Quantitation of arterial oxygen saturation (left) and respiratory rate (right) in P18 *Yap1*,*Wwtr1*^{iPCKO} and littermate control mice. Data represents mean± s.e.m. (n=12 controls and n=10 mutants, two-tailed unpaired t-test).

(**c**) Cell cycle analysis of cells from P7 *Yap1*,*Wwtr1*^{iPCKO} lung tissue by flow cytometry. After initial FSC/SSC gating, cells were subdivided into CD31^{+} EpCAM^{-} or CD31^{-} EpCAM^{+} subsets. For each subset, 2N, 4N and S-phase cells were quantified based on staining for EdU and DAPI.

(**d**) Maximum intensity projections of P7 *Yap1*,*Wwtr1*^{iPCKO} and littermate control lungs stained for active caspase 3 (green) and DAPI (blue). Scale bar, 50μm. Quantitation of active caspase 3 positive cells and total cells shown below. Data represents mean± s.e.m. (n=4 mice; two-tailed unpaired t-test).

(**e**) Gating strategy for the purification of three subpopulations from P7 and P12 *Yap1*,*Wwtr1*^{iPCKO} lung tissue in flow cytometry. After initial FSC/SSC gating and doublet exclusion, live cells were gated as DAPI^{-} CD45^{-} Ter119^{-}, and then subdivided in two steps into (i) EpCAM^{+} CD31^{-} PDGFRβ^{-} PDGFRα^{-}, (ii) EpCAM^{-} CD31^{-} PDGFRβ^{+} PDGFRα^{-} and (iii) EpCAM^{-} CD31^{+} PDGFRβ^{-} PDGFRα^{-} fractions.

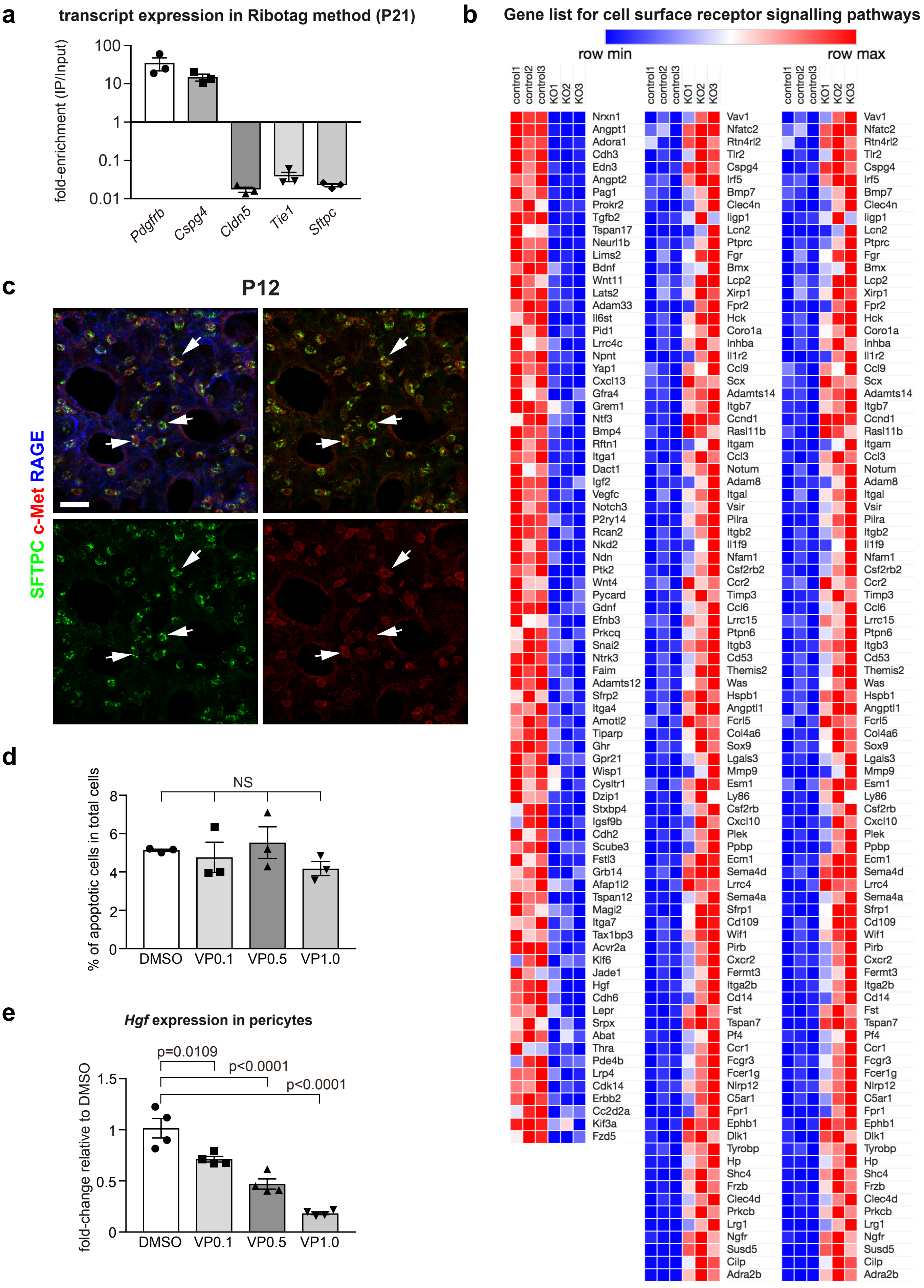
a
transcript expression in Ribotag method (P21)
fold-enrichment (IP/Input)
100
10
1
0.1
0.01
Pdgfrb
Cspg4
Cldn5
Tie1
Sftpc
b
Gene list for cell surface receptor signalling pathways
row min
row max
control1
control2
control3
KO1
KO2
KO3
Nrxn1 Angpt1 Adora1 Cdh3 Edn3 Angpt2 Pag1 Prokr2 Tgfb2 Tspan17 Neurl1b Lims2 Bdnf Wnt11 Lats2 Adam33 Il6st Pid1 Lrrc4c Npnt Yap1 Cxcl13 Gfra4 Grem1 Ntf3 Bmp4 Rftn1 Itga1 Dact1 Igf2 Vegfc Notch3 P2ry14 Rcan2 Nkd2 Ndn Ptk2 Wnt4 Pycard Gdnf Efnb3 Prkcq Snai2 Ntrk3 Faim Adamts12 Sfrp2 Itga4 Amotl2 Tiparp Ghr Gpr21 Wisp1 Cysltr1 Dzip1 Stxbp4 Igsf9b Cdh2 Scube3 Fstl3 Grb14 Afap1l2 Tspan12 Magi2 Itga7 Tax1bp3 Acvr2a Klf6 Jade1 Hgf Cdh6 Lepr Srpx Abat Thra Pde4b Lrp4 Cdk14 Erbb2 Cc2d2a Kif3a Fzd5
Vav1 Nfatc2 Rtn4rl2 Tlr2 Cspg4 Irf5 Bmp7 Clec4n Iigp1 Lcn2 Ptprc Fgr Bmx Lcp2 Xirp1 Fpr2 Hck Coro1a Inhba Il1r2 Ccl9 Scx Adamts14 Itgb7 Ccnd1 Rasl11b Itgam Ccl3 Notum Adam8 Itgal Vsir Pilra Itgb2 Il1f9 Nfam1 Csf2rb2 Ccr2 Timp3 Ccl6 Lrrc15 Ptpn6 Itgb3 Cd53 Themis2 Was Hspb1 Angptl1 Fcrl5 Col4a6 Sox9 Lgals3 Mmp9 Esm1 Ly86 Csf2rb Cxcl10 Plek Ppbp Ecm1 Sema4d Lrrc4 Sema4a Sfrp1 Cd109 Wif1 Pirb Cxcr2 Fermt3 Itga2b Cd14 Fst Tspan7 Pf4 Ccr1 Fcgr3 Fcer1g Nlrp12 C5ar1 Fpr1 Ephb1 Dlk1 Tyrobp Hp Shc4 Frzb Clec4d Prkcb Lrg1 Ngfr Susd5 Cilp Adra2b
c
P12
SFTPC c-Met RAGE
d
% of apoptotic cells in total cells
NS
8
6
4
2
0
DMSO
VP0.1
VP0.5
VP1.0
e
Hgf expression in pericytes
fold-change relative to DMSO
p=0.0109
p<0.0001
p<0.0001
1.5
1
0.5
0
DMSO
VP0.1
VP0.5
VP1.0

Supplementary Figure 6 Signalling pathway analysis of *Yap1,Wwtr1* mutant mice

(**a**) RT-qPCR analysis of indicated transcripts expression in input and immunoprecipitated (IP) RNA from P21 *Pdgfrb(BAC)-CreERT2 Rpl22HA* mice. Data represents mean± s.e.m. (n=3 mice). Note strong enrichment of pericyte markers (*Pdgfrb* and *Cspg4*) relative to input.

(b) Gene lists from Gene ontology cluster focussing on cell surface receptor signalling pathways.

(c) High magnification images of P12 lungs stained for SFTPC (green), c-MET (red) and RAGE (blue) related to Figure 4f. Arrows indicate SFTPC-positive c-MET+ cells. Scale bar, 30μm.

(**d**) Apoptotic signals from 0.1, 0.5 or 1μM Verteporfin (VP) treated cultured pericytes and controls at 48 hours using Annexin V and DAPI staining. Data represents mean± s.e.m. (n=3, one-way ANOVA with Tukey's multiple comparison *post hoc* test).

(**e**) RT-qPCR analysis of *Hgf* mRNA expression from 0.1, 0.5 or 1μM VP treated cultured pericytes and controls at 48 hours. Data represents mean± s.e.m. (n=4, one-way ANOVA with Tukey's multiple comparison *post hoc* test).

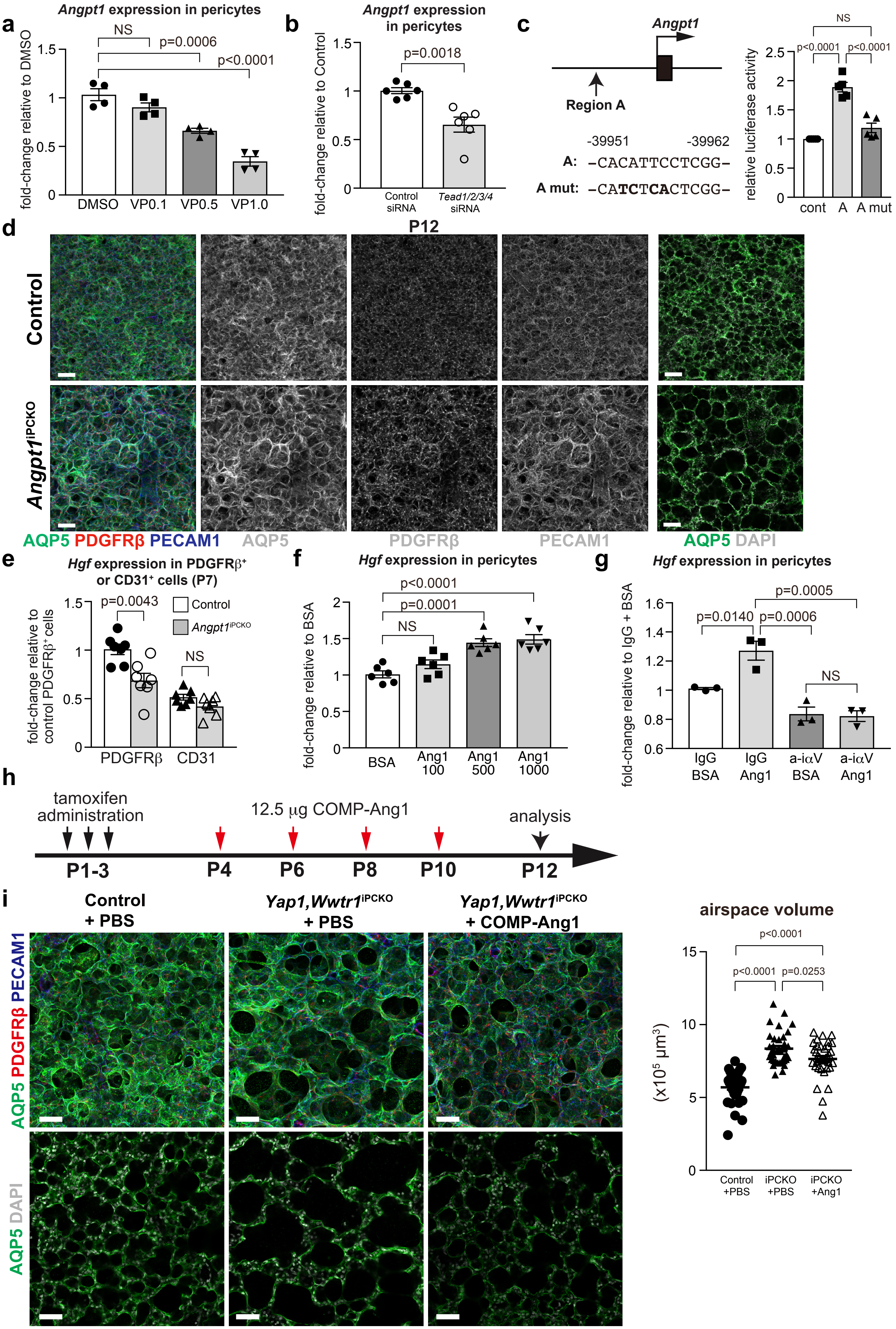

a
Angpt1 expression in pericytes
fold-change relative to DMSO
NS
p=0.0006
p<0.0001
DMSO
VP0.1
VP0.5
VP1.0
b
Angpt1 expression in pericytes
fold-change relative to Control
p=0.0018
Control siRNA
Tead1/2/3/4 siRNA
c
Angpt1
Region A
-39951
-39962
A: -CACATTCCTCGG-
A mut: -CATCTCACTCGG-
relative luciferase activity
NS
p<0.0001
p<0.0001
cont
A
A mut
d
P12
Control
Angpt1iPCKO
AQP5 PDGFRβ PECAM1
AQP5
PDGFRβ
PECAM1
AQP5 DAPI
e
Hgf expression in PDGFRβ+ or CD31+ cells (P7)
fold-change relative to control PDGFRβ+ cells
p=0.0043
NS
Control
Angpt1iPCKO
PDGFRβ
CD31
f
Hgf expression in pericytes
fold-change relative to BSA
p<0.0001
p=0.0001
NS
BSA
Ang1 100
Ang1 500
Ang1 1000
g
Hgf expression in pericytes
fold-change relative to IgG + BSA
p=0.0005
p=0.0140
p=0.0006
NS
IgG BSA
IgG Ang1
a-iαV BSA
a-iαV Ang1
h
tamoxifen administration
12.5 μg COMP-Ang1
analysis
P1-3
P4
P6
P8
P10
P12
i
Control + PBS
Yap1,Wwtr1iPCKO + PBS
Yap1,Wwtr1iPCKO + COMP-Ang1
AQP5 PDGFRβ PECAM1
AQP5 DAPI
airspace volume
(x10^5 μm^3)
p<0.0001
p<0.0001
p=0.0253
Control +PBS
iPCKO +PBS
iPCKO +Ang1

Supplementary Figure 7 Effect of Angpt1 in lung morphogenesis

(**a**) RT-qPCR analysis of *Angpt1* mRNA expression from 0.1, 0.5 or 1μM VP treated cultured pericytes and controls at 48 hours. Data represents mean± s.e.m. (n=4, one-way ANOVA with Tukey's multiple comparison *post hoc* test).

(**b**) Relative gene expression analysis by qPCR of *Angpt1* in siRNA-mediated knock down of *Tead1/2/3/4* or control pericytes. Data represents mean± s.e.m. (n=6; two-tailed unpaired t-test).

(**c**) Luciferase reporter assay for candidate binding region with either intact TEAD consensus motif or with mutations. Relative luciferase activity represents the ratio of Firefly and Renilla luciferase activity for each sample. Data represents mean± s.e.m. (n=5, one-way ANOVA with Tukey's multiple comparison *post hoc* test).

(**d**) Wide field view of 3D reconstruction confocal images (4 left panels) of P12 *Angpt1*^{iPCKO} (red) and PECAM1 (blue). Single optical section images (AQP5 (green) and DAPI (white)) are shown in the most right panels. Scale bar, 100μm.

(**e**) *Hgf* expression in freshly sorted PDGFRβ+ or CD31+ cells from P7 *Angpt1*^{iPCKO} and littermate control lungs. Data represents mean± s.e.m. (n=7 mice; NS: not significant, two-tailed unpaired t-test).

(**f**) RT-qPCR analysis of *Hgf* mRNA expression from 100, 500 or 1000 ng/ml COMP-Ang1 treated cultured pericytes and controls at 24 hours. Data represents mean± s.e.m. (n=6, one-way ANOVA with Tukey's multiple comparison *post hoc* test).

(**g**) *Hgf* expression in BSA (1000 ng/ml) or COMP-Ang1 (1000 ng/ml) treated pericytes in the presence of IgG (30 μg/ml) or antibody against Integrin Alpha V (a-iαV) 24 hours after each treatment. Data represents mean± s.e.m. (n=3, one-way ANOVA with Tukey's multiple comparison *post hoc* test).

(**h**) Scheme showing the time points of tamoxifen administration, COMP-Ang1 treatment and analysis for *Yap1*,*Wwtr1* ^{iPCKO} mice.

(**i**) 3D reconstruction confocal images (top panels) or single optical section images (bottom panels) of P12 *Yap1*,*Wwtr1*^{iPCKO} and littermate control lungs from mice treated with PBS or (red) and PECAM1 (blue) or AQP5 (green) and DAPI (white). Scale bar, 50μm. Quantitation of airspace volume shown on the right. Data represents mean± s.e.m. (n=6 mice, one-way ANOVA with Tukey's multiple comparison *post hoc* test).

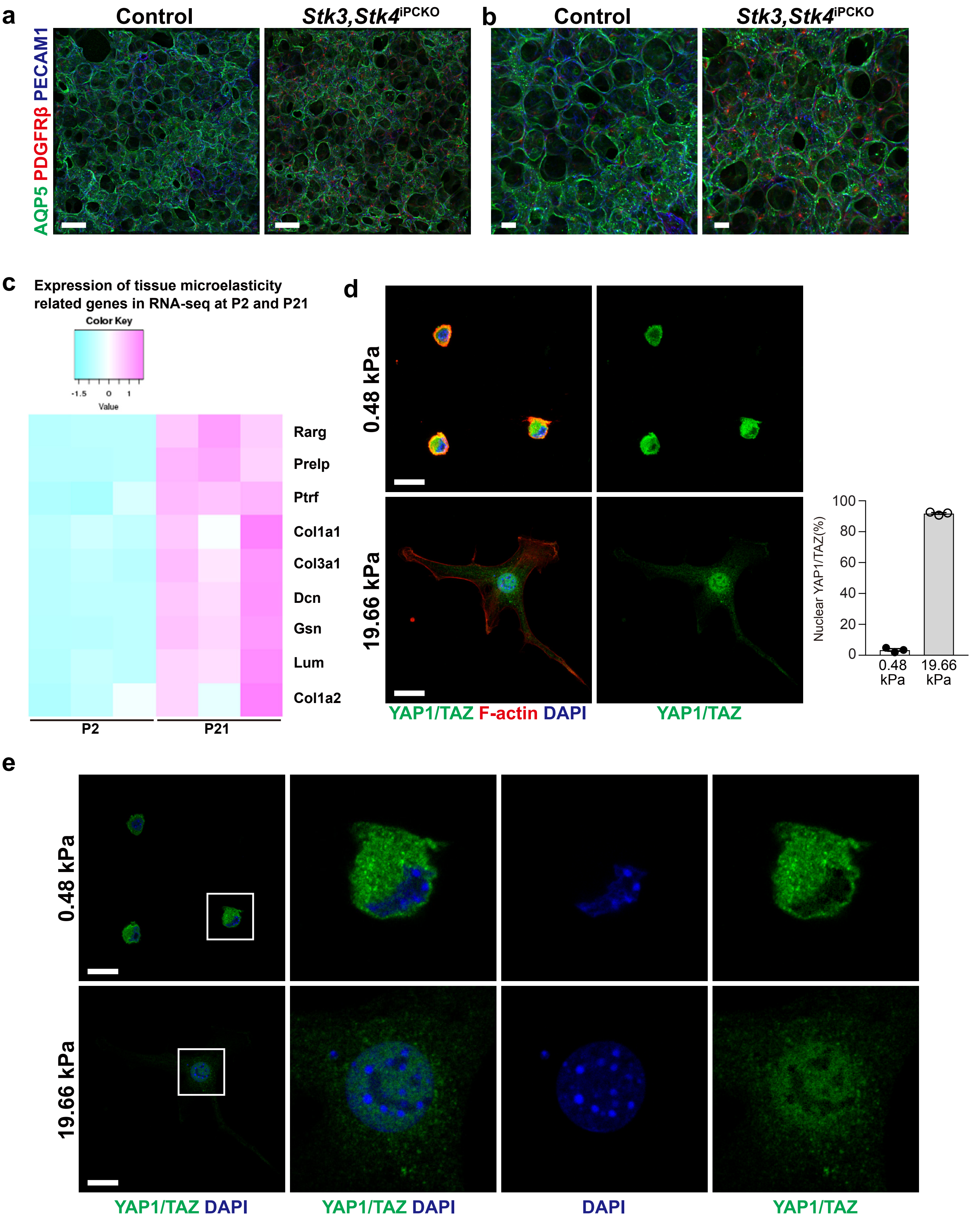
a
Control
Stk3,Stk4iPCKO
AQP5 PDGFRβ PECAM1
b
Control
Stk3,Stk4iPCKO
c
Expression of tissue microelasticity related genes in RNA-seq at P2 and P21
Color Key
-1.5
0
1
Value
Rarg
Prelp
Ptrf
Col1a1
Col3a1
Dcn
Gsn
Lum
Col1a2
P2
P21
d
0.48 kPa
19.66 kPa
YAP1/TAZ F-actin DAPI
YAP1/TAZ
Nuclear YAP1/TAZ(%)
100
80
60
40
20
0
0.48 kPa
19.66 kPa
e
0.48 kPa
19.66 kPa
YAP1/TAZ DAPI
YAP1/TAZ DAPI
DAPI
YAP1/TAZ

Supplementary Figure 8 ECM stiffness regulates YAP/TAZ localization

(**a**, **b**) 3D reconstruction confocal images of P12 *Stk3,Stk4*^{iPCKO} and littermate control lungs (red) and PECAM1 (blue). Panels in (**b**) show higher magnification. Scale bar, 50μm (**a**) and 15μm (**b**).

(**c**) Heat map of selected genes related to tissue microelasticity from RNA-seq analysis of P2 and P21 *Pdgfrb(BAC)-CreERT2 Rpl22HA* mice. Scale indicates expression relative (Z-score) to median of six samples.

(**d**) High-resolution confocal images of YAP1/TAZ (green), F-actin (red) and nuclei (DAPI) in pericytes cultured on stiff (elastic modulus of 19.66 kPa) and soft (0.48 kPa) fibronectin-coated hydrogels. Graphs indicate the percentage of cells with nuclear YAP1/TAZ (n=3 independent experiments). Scale bar, 20μm.

(**e**) Single optical section images shown in (**d**). Panels on the right show higher magnification of corresponding insets. Scale bar, 20μm.

Fig.3k

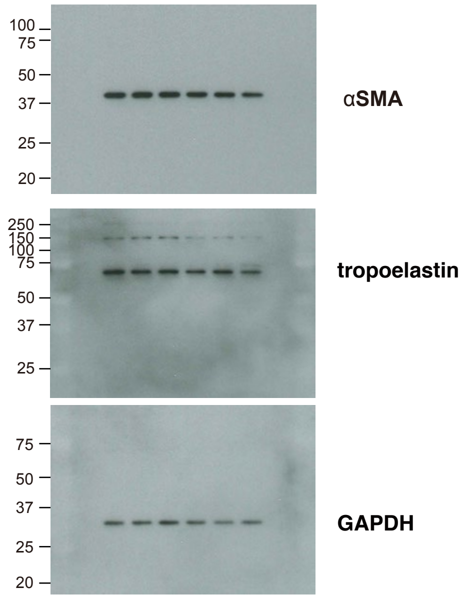

Fig.4h

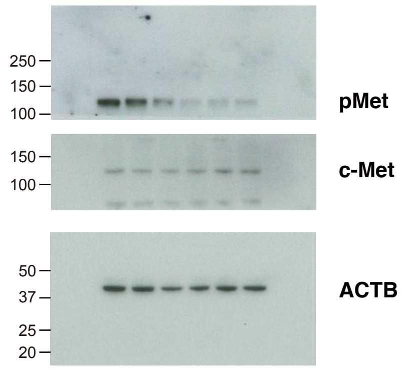

Fig.5f

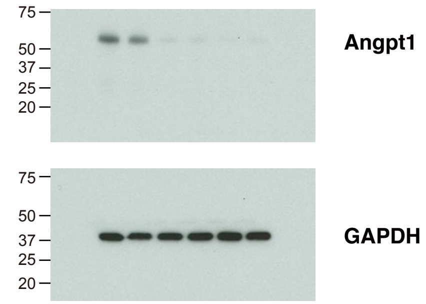

Fig.5g

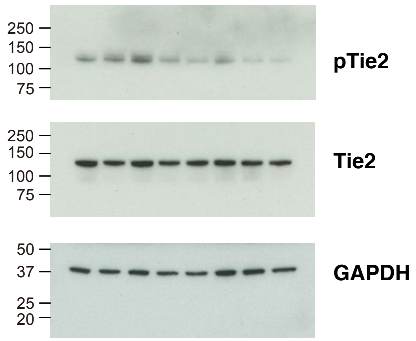

SFig.4g

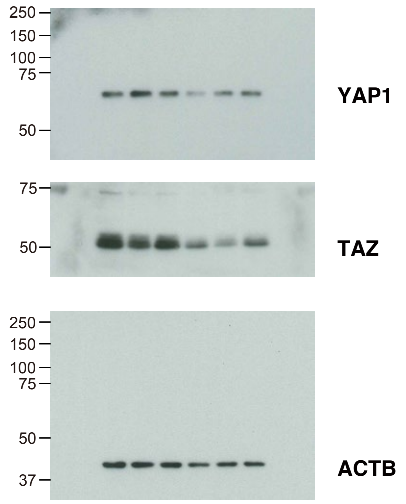

Supplementary Figure 9. Uncropped western blots related to Figs. 3, 4, 5 and Supplementary Figure 4
