# Supplementary material for: Pulmonary pericytes regulate lung morphogenesis
Source: Nat Commun. 2018 Jun 22;9:2448. doi: 10.1038/s41467-018-04913-2 (PMC6015030; doi:10.1038/s41467-018-04913-2)
Supplement: Supplementary file 3 — Description of Additional Supplementary Files [file 41467_2018_4913_MOESM3_ESM.pdf]

Description of Additional Supplementary Files

File Name: Supplementary Movie 1 / 2.

Description: Visualization of alveolar epithelial cells, pulmonary endothelial cells and pericytes. Three-dimensional reconstruction movie from confocal images with Airyscan detection showing AQP5-stained type 1 alveolar epithelial cells (green), PDGFRb-stained pulmonary pericytes (PCs) (red) and PECAM1-stained endothelial cells (ECs) (blue/white) in lung at 4 weeks shown in Fig. 1a. ECs (white) (Movie 1) and epithelial cells (green), PCs (red) and ECs (blue) (Movie 2)

File Name: Supplementary Movie 3 / 4.

Description: Visualization of pulmonary endothelial cells in P12. Yap1,Wwtr1iPCKO and littermate control lungs. Three-dimensional high magnification reconstruction videos of P12 Yap1,Wwtr1iPCKO (Movie 4) and littermate control (Movie 3) lungs stained for PECAM1 (white) shown in Fig. 3f.
